# Quantification of interventricular dyssynchrony by phase contrast magnetic resonance angiography

**DOI:** 10.1186/1532-429X-11-S1-P82

**Published:** 2009-01-28

**Authors:** Kai Muellerleile, Loant Baholli, Michael Groth, Katharina Koopmann, Achim Barmeyer, Ralf Koester, Gerhard Adam, Thomas Meinertz, Stephan Willems, Gunnar Lund

**Affiliations:** University Medical Center Hamburg-Eppendorf, Hamburg, Germany

**Keywords:** Ejection Fraction, Cardiac Resynchronization Therapy, Flow Curve, Pulmonary Valve, Left Bundle Branch Block

## Objective

Interventricular dyssynchrony is typically assessed by pulsed-wave echocardiography (PW-Echo) as the delay between onset of aortic and pulmonary flow. Recent multicenter trials demonstrated the value of this interventricular dyssychrony to predict response to cardiac resynchronization therapy (CRT). In the present study, the ability of phase contrast magnetic resonance angiography (PC-MRA) was assessed to quantify interventricular dyssynchrony in comparison with PW-Echo.

## Methods

40 patients with stable heart failure NYHA Class 2 to 3, reduced ejection fraction (28 ± 11%), with (n = 21) or without (n = 19) complete left bundle branch block were prospectively included. Transvalvular flow curves of the aortic and pulmonary valve were acquired by PC-MRA and PW-Echo. Interventricular delay was calculated for PC-MRA as the delay between onset of aortic and pulmonary flow in analogy to PW-Echo. Interventricular delays by PC-MRA were correlated with PW-Echo; agreement was assessed by Bland-Altman analysis.

## Results

**Figure 1:**
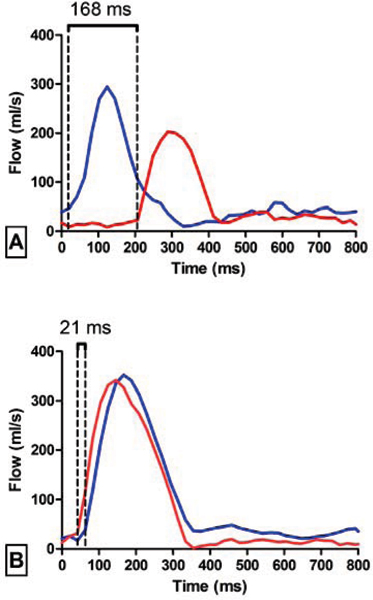
Figure 1

A strong correlation between interventricular delays by PW-Echo and PC-MRA was found (r = 0.89, P < 0.0001). Bland-Altman analysis demonstrated a good agreement between both methods (Mean difference -6 ± 15 ms). An example of the assessment of interventricular delays by PC-MRA in one patient with *(A)* and one patient without interventricular dyssynchrony *(B)* is illustrated by Figure 1. Aortic (red) and pulmonary (blue) valve flow curves are plotted; interventricular delay by PC-MRA was 168 ms in *patient A* (PW-Echo = 146 ms) and 21 ms in *patient B* (PW-Echo = 6 ms).

## Conclusion

PC-MRA quantifies interventricular dyssynchrony comparable with PW-Echo. PC-MRA has the potential to identify responders to CRT.

